# Coffee-Derived Phenolic Compounds Activate Nrf2 Antioxidant Pathway in I/R Injury In Vitro Model: A Nutritional Approach Preventing Age Related-Damages

**DOI:** 10.3390/molecules27031049

**Published:** 2022-02-03

**Authors:** Elena Lonati, Tatiana Carrozzini, Ilaria Bruni, Pedro Mena, Laura Botto, Emanuela Cazzaniga, Daniele Del Rio, Massimo Labra, Paola Palestini, Alessandra Bulbarelli

**Affiliations:** 1 School of Medicine and Surgery, University of Milano-Bicocca, 20900 Monza, Italy; elena.lonati1@unimib.it (E.L.); t.carrozzini@campus.unimib.it (T.C.); laura.botto@unimib.it (L.B.); emanuela.cazzaniga@unimib.it (E.C.); paola.palestini@unimib.it (P.P.); 2 Bicocca Center of Science and Technology for Food, University of Milano-Bicocca, 20126 Milano, Italy; ilaria.bruni@unimib.it (I.B.); massimo.labra@unimib.it (M.L.); 3 Department of Biotechnology and Biosciences, University of Milano-Bicocca, 20126 Milano, Italy; 4 Human Nutrition Unit, Department of Food and Drug, University of Parma, 43124 Parma, Italy; pedromiguel.menaparreno@unipr.it (P.M.); daniele.delrio@unipr.it (D.D.R.); 5 School of Advanced Studies on Food and Nutrition, University of Parma, 43121 Parma, Italy

**Keywords:** coffee phytoextract, coffee metabolites, phenolic compounds, oxidative stress, Nrf2/ARE antioxidant pathway, oxygen and glucose deprivation, ischemia, rat brain endothelial cells

## Abstract

Age-related injuries are often connected to alterations in redox homeostasis. The imbalance between free radical oxygen species and endogenous antioxidants defenses could be associated with a growing risk of transient ischemic attack and stroke. In this context, a daily supply of dietary antioxidants could counteract oxidative stress occurring during ischemia/reperfusion injury (I/R), preventing brain damage. Here we investigated the potential antioxidant properties of coffee-derived circulating metabolites and a coffee pulp phytoextract, testing their efficacy as ROS scavengers in an in vitro model of ischemia. Indeed, the coffee fruit is an important source of phenolic compounds, such as chlorogenic acids, present both in the brewed seed and in the discarded pulp. Therefore, rat brain endothelial cells, subjected to oxygen and glucose deprivation (OGD) and recovery (ogR) to mimic reperfusion, were pretreated or not with coffee by-products. The results indicate that, under OGD/ogR, the ROS accumulation was reduced by coffee by-product. Additionally, the coffee extract activated the Nrf2 antioxidant pathway via Erk and Akt kinases phosphorylation, as shown by increased Nrf2 and HO-1 protein levels. The data indicate that the daily intake of coffee by-products as a dietary food supplement represents a potential nutritional strategy to counteract aging.

## 1. Introduction

The worldwide population is becoming increasingly elderly due to the improvement of medical care. Nevertheless, this is accompanied by age-related injury processes often connected to bioenergetic impairments as well as alterations in the reduction–oxidation (redox) homeostasis. Oxidative Stress is specifically defined as “a disturbance in the prooxidant-antioxidant balance in favor of the former” [1,2]. In aged-related disease, the upregulation of free radical oxygen species (ROS) generation overcomes the neutralizing capacity of the endogenous antioxidant defense system [3]. This unbalance is associated with risk factors of cerebral vascular disease, such as obesity, diabetes, hypertension, and environmental pollution [4,5]. In this scenario, the transient ischemic attack (TIA) represents a medical emergency since it increases the risk of subsequent ischemic stroke. The ischemia/reperfusion (I/R) injury leads to blood–brain barrier (BBB) damage, intracellular Ca^2+^ accumulation, cell death or apoptosis, inflammation, and oxidative stress, with an increased risk of developing brain damage [6]. Recurrent TIA can occur without being diagnosed until the appearance of a serious event associated with a focal neurologic deficit and speech disturbance [7]. For that reason, the elderly population needs to be educated about the importance of blood pressure control, discontinuing smoking, and eating a healthy diet. Indeed, a nutritional protocol aimed at the daily supply of essential and nonessential antioxidant nutrients can contribute to counteracting the oxidative impacts in aged-related processes [8] and may be a promising neuroprotective strategy [9]. Most antioxidant nutrients can be obtained through the intake of specific foods rich in natural exogenous antioxidants, such as vitamin E, vitamin C, carotenoids, omega-3/omega-6 fatty acids, and (poly)phenols [10,11]. (Poly)phenols are a group of biologically active compounds with well-recognized beneficial effects against free radicals [12]. They occur in fruits, vegetables, grain, tea, and coffee. Considering that over 500 billion cups of coffee are consumed worldwide on an annual basis [13], coffee represents a major source of (poly)phenols [14,15]. The main phenolic compounds contained in the beverage are hydroxycinnamates, in the form of chlorogenic acids (CGAs) [16]. Following consumption of coffee, some of the CGAs are hydrolyzed by intestinal and hepatic enzymes forming several phenolic metabolites, principally derivatives of caffeic and ferulic acids, which appear in the circulatory system alongside smaller amounts of unmodified CGAs [16,17]. The beneficial properties of coffee are related to the high CGA content, which is a potent free radical scavenger and an inducer of the nuclear factor erythroid 2-related factor 2 (Nrf2)/antioxidant response element (ARE) signaling pathway [9]. CGAs are also present with significant quantities in the pulp of the coffee fruit.

In the commercial production of coffee from Coffea beans, an enormous amount of waste CGA-containing pulp is generated [18]. Modern bioeconomic value chain guidelines propose that the discarded pulp could be reutilized in pharmaceutical, cosmetic, and feed industries [19]. Nowadays, the pulp, like other waste parts of the fruit, is used as an organic component of fertilizers for the organic cultivation of coffee plantations. However, thanks to the bioactive properties of discovered molecules in the pulp [20], it has been proposed to use coffee pulp by-products as ingredients in foods for human nutrition [21]. In this context, it is of interest that a phytoextract (Phyt) obtained from Coffea Arabica pulp has been reported to have anti-inflammatory activity in gastric epithelial cells [22]. Among molecules contained in the Phyt, Magoni and colleagues identified several quinic acids and caffeic acid derivatives that are known to exert a good antioxidant activity alone or also in a mixture [23]. They also detected the presence of procyanidin dimers and trimers type A, a specific group of polyphenols, which have a large number of bioactive properties [24].

The HPLC characterization of Phyt highlights its potential antioxidant power; thus, in the current study, we tested coffee by-product’s efficacy as ROS scavengers under I/R injury. The analyses were carried out in rat brain endothelial cells (RBE4) subjected to oxygen and glucose deprivation (OGD) followed by nutrients, glucose, and oxygen recovery (ogR) to mimic reperfusion. It was shown that under OGD/ogR, ROS accumulation is dampened by pretreatment with CGA-derived metabolites as well the coffee pulp phytoextract. The potential of the Phyt as an activator of the endogenous antioxidant Nrf2/ARE pathway was also investigated in order to understand its involvement in the antioxidant defenses. Data here presented supports the importance of waste material recovery along the agro-industrial coffee supply chain, highlighting coffee pulp as a promising source of antioxidants.

## 2. Results and Discussion

### 2.1. Coffee Metabolites Antioxidant Effect under Conditions Mimic Ischemia

In the last decades, a healthy lifestyle has become a key approach to counteract the inflammation and the oxidative stress characterizing several acute and chronic diseases. Several epidemiological studies in the nutrition field have revealed that (poly)phenol-rich diets can provide beneficial effects in humans, helping in the prevention of cognitive decline and degenerative disorders [25,26]. Indeed, growing evidence highlights the important role of antioxidant-rich foods as scavengers of free radicals in those conditions characterized by an imbalance between the production of oxidant molecules and the antioxidant defenses. More recently, coffee has been described as a very important source of dietary antioxidant compounds [27], exerting a protective action against the reactive oxygen species (ROS). The antioxidant power of coffee might be a resource to prevent oxidative damages induced by cerebral ischemic events, often silent and not diagnosed until potentially life-threatening symptoms become apparent. Several repeated ischemia leads to progressive loss of blood–brain barrier function and the consequent alterations in the brain regions. The sudden reoxygenation, also called “reperfusion damage”, generates ROS triggering dangerous pro-oxidant mechanisms [28].

Previous studies have demonstrated the neuroprotective effect of caffeic acid (CA) on focal cerebral ischemia–reperfusion injury [29,30]. Chlorogenic acids (CGAs) are the most abundant phenolic compounds of coffee, and following ingestion, they undergo an extensive metabolic transformation by intestinal microbiota [31] and hepatic phase II conjugation enzymes [17]. Consequently, colonic metabolites are generally identified as sulfate, glucuronide, and methyl conjugates of caffeic acid, and they show longer elimination half times compared with metabolites absorbed in the upper gastrointestinal tract [16]. As a result, substantial amounts of CA derivatives are found in the plasma of coffee drinkers [17,32,33].

**Figure 1:**
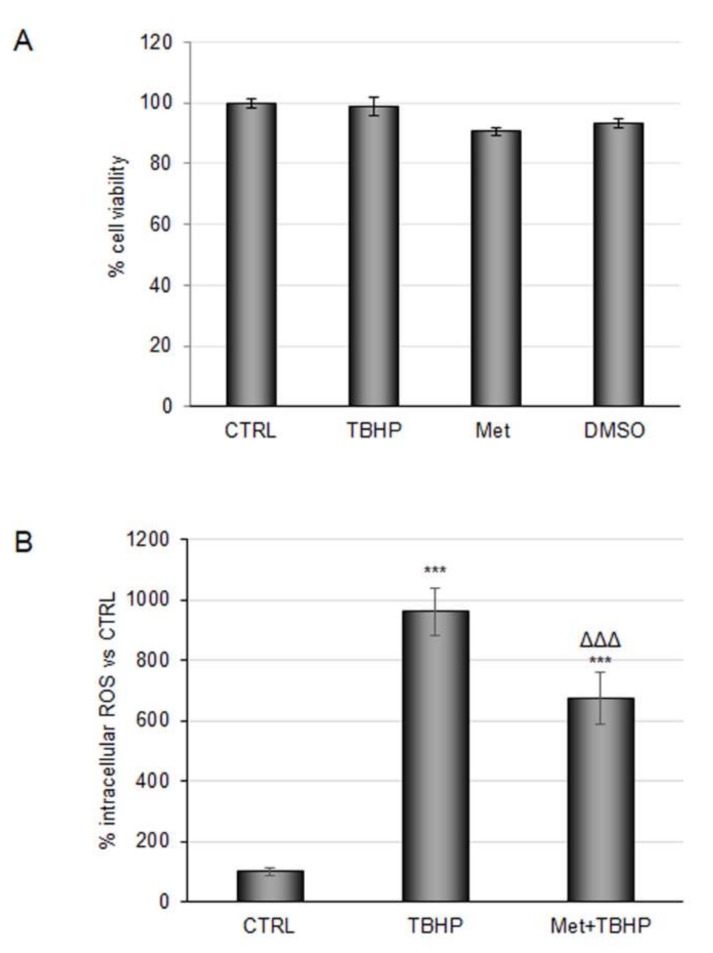
Evaluation of coffee-derived circulating metabolite antioxidant properties against TBHP in RBE4 cells. (**A**) RBE4 cells were treated with 100 nM of coffee-derived circulating metabolites for 48 h or treated with *tert*-Butyl hydroperoxide (TBHP) 200 μM for three hours; then, the MTT assay was performed to evaluate cell viability. Dimethyl sulfoxide (DMSO) (as a solvent to dissolve Metabolites) was also evaluated. (**B**) Reactive oxygen species (ROS) intracellular evaluation by 2,7-dichlorofluorescein (DCF) fluorescence intensity of cells treated with TBHP 200 μM for three hours alone or in combination with 100 nM metabolite mix pretreatment. The histograms, obtained from three distinct experiments, are expressed as a percentage of cell viability or intracellular ROS ± S.E. *** *p* < 0.001 versus CTRL (untreated cells); ᐃᐃᐃ *p* < 0.001 versus TBHP (positive control).

In a recent study, the profile of circulating phenolic metabolites was determined after repeated daily doses of coffee, and this revealed an estimation of 40–70% coffee CGAs bioavailability, with their metabolites entering the circulatory system [16,17]. Thus, we planned to explore the antioxidant effect of CGA-derived circulating metabolites in an in vitro model of ischemia. Based on the definition of daily average concentrations detected in individuals drinking about 3 cups of coffee daily, and to mimic a more physiological scenario, the antioxidant power of main CGA-derived metabolites were evaluated at a concentration of 100 nM, either alone or combined in a mixture. First, RBE4 cell viability was evaluated after 48 h of treatment. MTT assay confirmed the safety of the compounds employed (Figure 1A). Then, the antioxidant power of the mixture at the selected concentration was tested on cells subjected to *tert*-butyl hydroperoxide solution (TBHP), a well-known pro-oxidant, at 200 μM for 3 h. Dose and time of TBHP treatment were chosen based on experimental evidence described in Section 3. None of the individual metabolites promoted a reduction in the intracellular ROS amount compared with TBHP treatment (data not shown); conversely administration of the metabolite mixture induced a reduction of about 30%, suggesting a synergic effect of the test compounds (Figure 1B).

**Figure 2:**
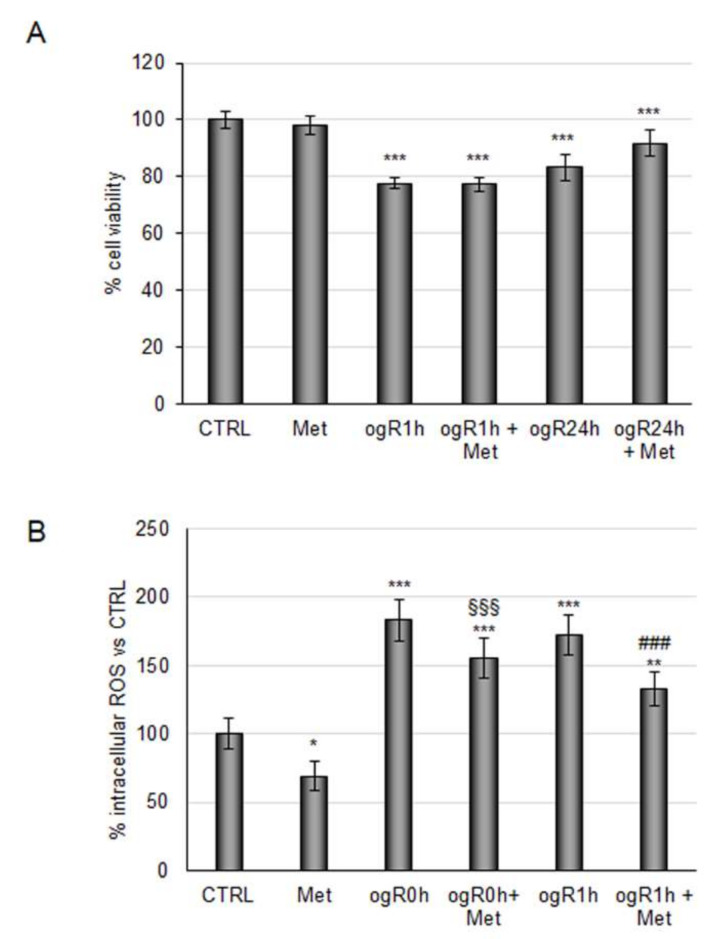
Effects of coffee-derived circulating metabolites against OGD/ogR-mediated oxidative stress. RBE4 cells pretreated or not for 24 h with 100 nM coffee-derived metabolites were subjected to oxygen and glucose deprivation and subsequent restoration of normoxic and normoglucidic conditions OGD/ogR exposure (**A**) Cell viability evaluated by MTT assay. (**B**) ROS intracellular evaluation by DCF fluorescence intensity. The histograms, obtained from three distinct experiments, are expressed as a percentage of cell viability or intracellular ROS as mean ± S.E. * *p* < 0.05, ** *p* < 0.01, *** *p* < 0.001 versus CTRL (untreated cells); §§§ *p* < 0.001 versus ogR0h; ### *p* < 0.001 versus to ogR1h.

In light of these results, the metabolite mixture was tested under conditions mimicking I/R injury. RBE4 cells pretreated with metabolites (100 nM) for 24 h were subjected to 3 h of oxygen and glucose deprivation (OGD). Immediately after deprivation, nutrients and oxygen were reinstated in order to mimic the reperfusion phase (ogR). Cell viability was analyzed at 1 h and 24 h after recovery, showing a 20% decrease at ogR1h, which was slightly recovered after 24 h (ogR24h) when cells were pretreated with metabolite mix (Figure 2A). According to Li and colleagues, who identified the highest ROS production within one hour after normoxic and normoglucidic conditions re-establishment, ogR0 and ogR1 h time points were chosen for measuring ROS intracellular levels [34]. Metabolite pretreatment reduced ROS levels of about 16% at time 0 and about 25% 1 h after recovery, demonstrating their antioxidant properties against the oxidative stress induced by reperfusion (Figure 2B). These data, in line with a recent study revealing the capability of CGA-derived metabolites as ROS scavengers [35], encourages the use of coffee products to dampen ROS enhancement under oxidant conditions.

### 2.2. Evaluation of Antioxidant Power of Coffee Pulp Phytoextract under Conditions Mimic Ischemia

A previous study on the anti-inflammatory property of Arabica coffee disclosed that a pulp extract was more active than seeds in preventing gastric epithelium inflammation [22]. Analysis of the pulp extract by LC-MS identified several (poly)phenols, most notably CGAs and CA derivatives and also procyanidin dimers and trimers. Considering that CGAs undergo rapid degradation during the roasting of coffee beans roasting, pulp phytoextract (Phyt) results in a better source of these compounds [36]. During the industrial processing of coffee beans, a large amount of pulp is discarded. Thus, the transformation of waste into valuable resources such as bioactive phytoextract for the food and pharmaceutical industry should be one of the goals of modern agricultural sustainability guidelines [37].

**Figure 3:**
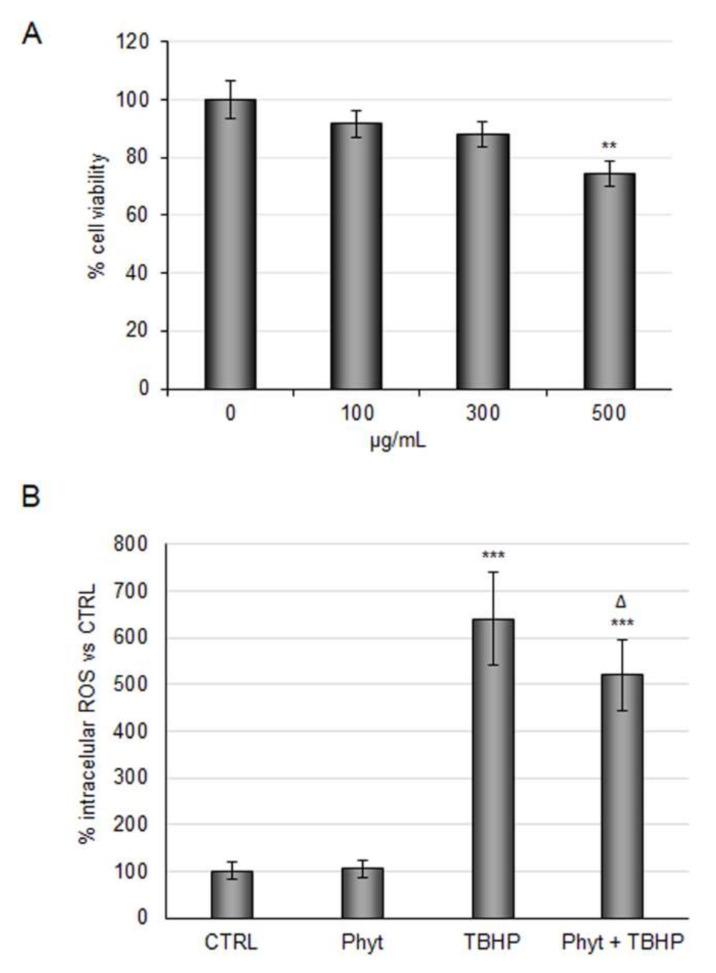
Evaluation of coffee pulp phytoextract antioxidant properties against TBHP in RBE4 cells. (**A**) RBE4 cells were treated with different concentrations (100, 300, 500 µg/mL) of phytoextract (Phyt) for 48 h. Then the MTT assay was performed to evaluate cell viability. (**B**) Intracellular ROS evaluation by DCF fluorescence intensity in RBE4 treated with TBHP 200 μM for three hours alone or in combination with 100 μg/mL pretreatment of Phyt. Phyt treatment was also tested. The histograms, obtained from three distinct experiments, are expressed as a percentage of cell viability or intracellular ROS as mean ± S.E. ** *p* <0.01, *** *p* < 0.001 versus CTRL (untreated cells); ᐃ *p* < 0.05 compared to TBHP.

Against this background, we evaluated the antioxidant power of the Phyt obtained by discarded Arabica coffee pulp. In order to test phytoextract safety and efficacy in our cellular model, increasing doses of the Phyt from 100 to 500 μg/mL were tested. The experiments showed a dose-dependent decrease of RBE4 cells viability (Figure 3A). Therefore, considering that anti-inflammatory activity has already been observed at a threshold dose of 100 μg/mL, the same concentration was assayed to test the Phyt antioxidant power. As shown in Figure 3B, Phyt administration alone did not impact intracellular ROS under basal conditions, while it reduced ROS increase by ~20% in cells treated with TBHP. Thus, the coffee pulp phytoextract exerted both antioxidant and anti-inflammatory activity.

**Figure 4:**
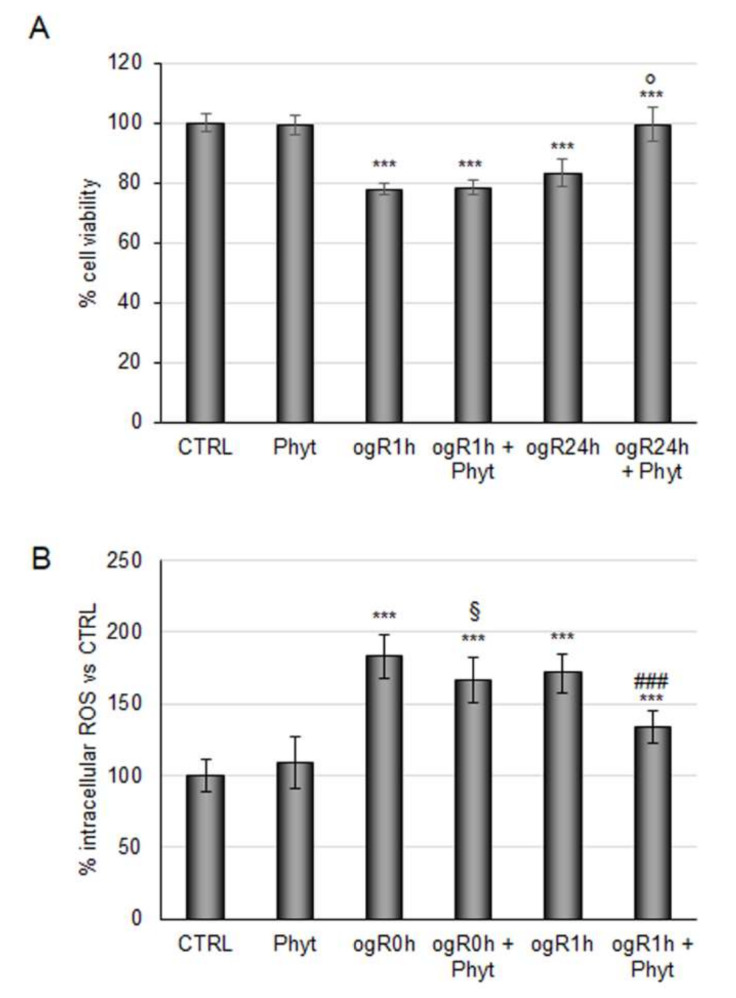
Effects of coffee pulp phytoextract against OGD/ogR-mediated oxidative stress. (**A**) RBE4 cells pretreated or not for 24 h with 100 μg/mL Phyt were subjected to OGD/ogR exposure (**A**) Cell viability evaluated by MTT assay (**B**) ROS intracellular evaluation by DCF fluorescence intensity. The histograms, obtained from three distinct experiments, are expressed as a percentage of cell viability or intracellular ROS as mean ± S.E. *** *p* < 0.001 versus CTRL (untreated cells); § *p* < 0.05 versus ogR0h; ### *p* < 0.001 versus ogR1h; *p* < 0.05 versus ogR24h.

This Phyt might strengthen the antioxidant defenses in ischemic conditions as assayed for metabolite blend. Thus, RBE4 cells subjected to OGD/ogR were pretreated with 100 μg/mL Phyt for 24 h. Coffee pulp phytoextract pretreatment did not revert the decrease in cell viability induced by OGD, but it was sufficient to recover cell viability during the following 24 h (Figure 4A). In parallel, Phyt treatment showed significant efficacy in ROS reduction (25%) at ogR1h (Figure 4B). Data obtained revealed the potential protective role of coffee pulp phytoextract against the intracellular free radical increase generated in a short time of reperfusion in BBB cells, leading to cell viability improvement in the long term. This evidence is consistent with positive results obtained by the employment of other (poly)phenols-enriched phytoextracts in OGD treatment [38].

### 2.3. Coffee Pulp Phytoextract Induced the Nrf2 Antioxidant Pathway under OGD/ogR

**Figure 5:**
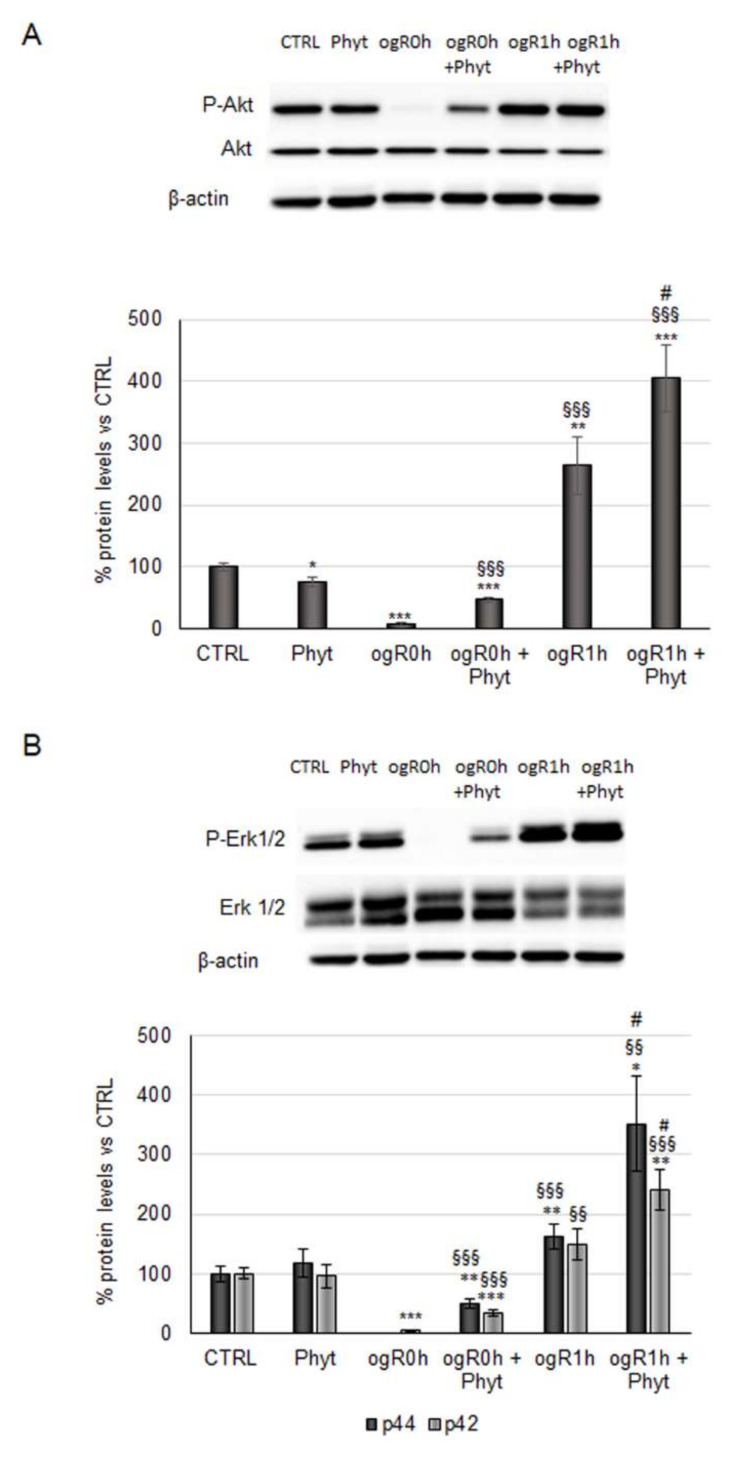
Effect of coffee-pulp phytoextract on Akt and Erk1/2 activation. Cells pretreated or not for 24 h of 100 μg/mL Phyt and then subjected to OGD/ogR exposure were harvested in a lysis buffer. Equal amounts of homogenate samples (as protein) were analyzed by SDS-PAGE electrophoresis and Western blotting. (**A**) p-Akt, akt and (**B**) p-Erk1/2, Erk1/2 were detected with specific antibodies and revealed by enhanced chemiluminescence (ECL). Samples were normalized on β-actin immunoreactivity. Histograms, obtained from three distinct experiments, represent the percentage of protein levels with respect to control as mean ± S.E. * *p* < 0.05, ** *p* < 0.01, *** *p* < 0.001 versus CTRL (untreated cells); §§ *p* < 0.01, §§§ *p* < 0.001 versus ogR0h; # *p* < 0.05 versus ogR1h.

It is noteworthy that the CGA antioxidant activity occurs as a combination of its direct ROS-quenching mechanism and the induction of endogenous antioxidant enzymes via activation of the transcription factor nuclear factor erythroid 2-related factor 2 (Nrf2)/antioxidant response element (ARE) signaling pathway. ROS might be further neutralized through the same endogenous antioxidant defenses induced by Nrf2 [9]. Indeed, Nrf2 upregulates a set of genes codifying enzymes involved in detoxification and elimination of reactive oxidants. According to the literature, the Nrf2 pathway might play a key role in cell survival processes against ischemia/reperfusion (I/R) injury [39]. Therefore, its activation was investigated in our in vitro model, analyzing, first, the kinases involved in the steadiness of the transcription factor. Nrf2 continuously undergoes proteasome degradation after Keap-1 binding, but under pro-oxidant stimuli, it is phosphorylated and detached from Keap-1 in order to migrate to the nucleus [40,41]. Akt and Erk1/2 kinases inhibition reduces the translocation and the nuclear accumulation of Nrf2 [41,42], suggesting a key role of these proteins in its regulation. Results in Figure 5A,B show that immediately after reoxygenation (ogR0h), the phosphorylation state of both kinases was reduced by ~90–95% in nonpretreated cells compared with the control. During OGD, the cells enter energy deficiency, and thus phosphorylation processes are inhibited. Moreover, ischemic conditions inhibit the usual cell energetic metabolisms and promote the autophagy pathway to recycle cellular components and damaged organelles for self-sustaining [43,44]. In the first hour of reperfusion, however, the kinase phosphorylation state significantly increased (163% Akt; 50% and 62% for p42 and p44 respectively), probably due to the restored availability of energy sources (Figure 5).

Interestingly, the Phyt pretreatment triggers phosphorylation of Akt and Erk1/2 both at ogR0h and ogR1h. Considering that during OGD, the kinases phosphorylation is almost completely inhibited, Phyt pretreatment exerts a strong effect at this time point. In the reperfusion phase, where the phosphorylation mechanisms are already restored by the availability of energy sources, a significant increment of phosphorylation state was detected. In particular, Phyt promoted a significant increase of pAkt^Ser473^/Akt ratio during OGD of about 6-fold (ogR0h + Phyt) and 50% after 1 h of reperfusion (ogR1h + Phyt) compared with nonpretreated cells (Figure 5A). In parallel, both Erks kinases phosphorylation was augmented in cells pretreated, as well as detected for Akt, showing a higher effect on p42 (Erk2) at ogR0h and on p44 (Erk1) at ogR1h. The phosphorylation ratio of p42 augmented 6-fold at ogR0h, while the p44 phosphorylated form appeared. At ogR1h, the p-p44 and p-p42 increased about 116% and 60%, respectively, compared with nonpretreated cells (Figure 5B). Although the differences in isoforms increase, a recent thorough review concludes that Erk1/2 are functionally redundant and act in a complex interplay in survival mechanisms [45]. Data obtained suggest that Phyt components could specifically activate survival signaling pathways overcoming the energy deficit.

**Figure 6:**
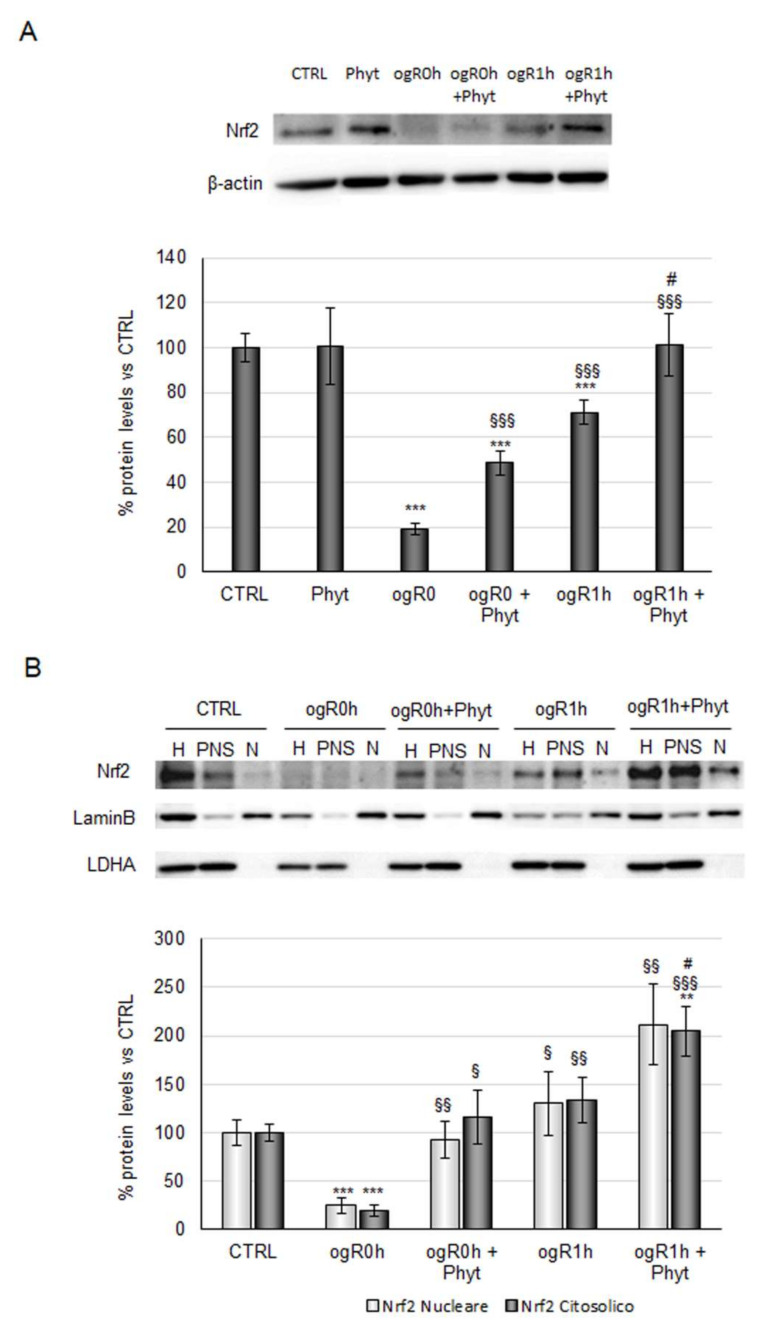
Effect of coffee-pulp phytoextract on Nrf2 protein levels and cellular localization. (**A**) Cells pretreated or not for 24 h with 100 μg/mL Phyt and then subjected to OGD/ogR exposure were harvested in lysis buffer. Equal amounts of homogenate samples (as protein) were analyzed by SDS-PAGE electrophoresis and Western blotting. Nrf2 was detected with a specific antibody and revealed by enhanced chemiluminescence (ECL). Samples were normalized on β-actin immunoreactivity. (**B**) Cells pretreated or not for 24 h of 100 μg/mL Phyt and then subjected to OGD/ogR exposure were harvested in PBS solution and then subjected to fractionation protocol. Homogenate, nuclear, and cytoplasmic fractions of each sample were analyzed by SDS-PAGE electrophoresis and Western blotting. Nrf2, LaminB, and LDH were detected with specific antibodies and revealed by enhanced chemiluminescence (ECL). Nuclear Nrf2 and cytoplasmatic Nrf2 were normalized on LaminB and LDH immunoreactivity, respectively. Histograms, obtained from three distinct experiments, represent the percentage of protein levels with respect to control as mean ± S.E. ** *p* < 0.01, *** *p* < 0.001 versus CTRL (untreated cells); § *p* < 0.05, §§ *p* < 0.01, §§§ *p* < 0.001 versus ogR0h; # *p* < 0.05 versus ogR1h.

Based on the Nrf2-kinases relationship observed in previous studies [40,46], the transcription factor protein levels were then investigated. Nrf2 protein levels followed the kinases trend, with a significant reduction of about 80% during OGD and a strong increase at ogR1h (270% with respect to ogR0h). As revealed for kinases, coffee pulp Phyt pretreatment is needed to enhance the cellular response at both the time point evaluated (150% at ogR0h and 40% at ogR1h with respect to cells only subjected to OGD/ogR treatment) (Figure 6A). Moreover, Nrf2 nuclear and cytoplasmic localization analysis showed an equal distribution between the two cellular fractions, with a trend that reflects the one observed in total homogenate (Figure 6B).

**Figure 7:**
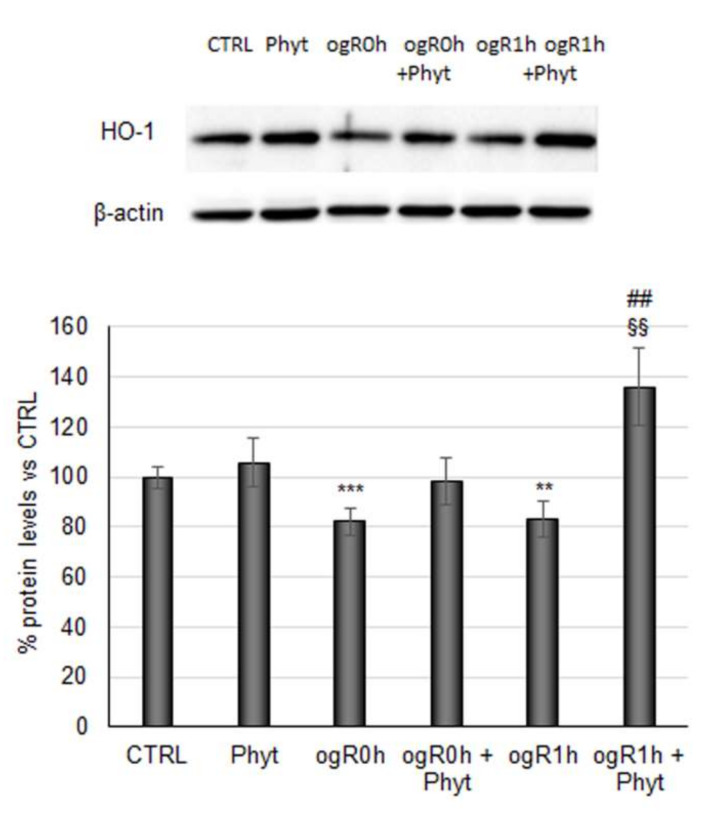
Effect of coffee pulp phytoextract on HO-1 protein levels. Cells pretreated or not for 24 h with 100 μg/mL Phyt and then subjected to OGD/ogR exposure were harvested in a lysis buffer. Equal amounts of homogenate samples (as protein) were analyzed by SDS-PAGE electrophoresis and Western blotting. HO-1 was detected with a specific antibody and revealed by enhanced chemiluminescence (ECL). Samples were normalized on β-actin immunoreactivity. Histograms, obtained from three distinct experiments, represent the percentage of protein levels with respect to control as mean ± S.E. ** *p* < 0.01, *** *p* < 0.001 versus CTRL; §§ *p* < 0.01 versus ogR0h; ## *p* < 0.01 versus ogR1h.

The Phyt, hence, induced the nuclear Nrf2 fraction augment both at ogR0h and ogR1h, compared with nuclear protein levels in cells subjected to the only OGD/ogR treatment, suggesting the presence of a major functionally active transcription factor. As mentioned above, Nrf2 stabilization in the nucleus results in the expression of antioxidant enzymes directly involved in the intracellular ROS neutralization [47]. Among them, the heme oxygenase-1 protein (HO-1) performs an essential role in opposing oxidative stress also under ischemic conditions [48,49]. Under OGD, HO-1 levels decreased by about 20% while they were completely rescued by pretreatment with Phyt, suggesting that Nrf2 nuclear translocation sustained the antioxidant enzyme expression. Interestingly, a significant 60% increase in HO-1 levels was detected at ogR1h in pretreated cells (ogR1h + Phyt) with respect to the ones exposed only to OGD/ogR, in which the levels remain almost stable (Figure 7).

HO-1 profile activation correlated with attenuation of ROS accumulation, suggesting the significance of its role among the endogenous enzymatic processes driven to ROS neutralization.

The observed activation of the Nrf2/ARE pathway was probably due to the presence of quinic acid derivatives, and procyanidins dimers and trimers type A in the coffee pul phytoextract. Indeed, individually taken, they are good enhancers of Nrf2 activity, incrementing its nuclear localization and the gene targets expression (such as HO-1). Moreover, quinic acid derivatives are also efficient ROS scavengers against TBHP-mediated oxidative stress, while procyanidins exert anti-inflammatory activity downregulating the intracellular inflammatory mechanisms with the consequent decrease in ROS generation [50,51,52].

## 3. Materials and Methods

### 3.1. Materials

All powdered reactants, *tert*-Butyl hydroperoxide (TBHP), 2,7-dichlorofluorescein diacetate (DCFH-DA) probe, 3-2,5-diphenyltetrazolium bromide (MTT), Dimethyl sulfoxide (DMSO), and Nuclei EZ Lysis Buffer were from Sigma Aldrich (Milano, Italy). The 5% CO_2_:95% N_2_ gas cylinder was from Sapio (Monza, Italy). Collagen I rat tail solution for RBE4 cell culture, and Novex Shasp Pre-Stained were obtained from Invitrogen, Life Technologies Italia (Monza, Italy).

All stock solutions for RBE4 cell culture, including alpha-MEM medium, Ham’s F-10 nutrient medium, and geneticin solution antibiotic, l-Glutamine, Penicillin/Streptomycin, and Fetal Bovine Serum (FBS), were purchased from Euroclone (Milano, Italy). The complete protease inhibitor cocktail was supplied by Roche Diagnostics S.p.A (Milano, Italy). Anti-Erk 1-2, anti-P-Erk 1-2, anti-HO-1 antibodies were from Santa Cruz Biotechnology (Santa Cruz, CA, USA). Anti-Akt, anti-P-Akt, anti-LDH, and anti-LaminB antibodies were acquired from Cell Signaling Technology (Danvers, MA, USA). Secondary horseradish peroxidase (HRP)-conjugated antibodies and enhanced chemiluminescence (ECL) SuperSignal detection kit were purchased from Pierce (Rockford, IL, USA). Coffee metabolites and phytoextract derived from coffee processing waste were supplied, respectively, by Prof. Daniele Del Rio (University of Parma) and Prof. Massimo Labra (University of Milano-Bicocca).

### 3.2. Rat Brain Endothelial Cell Line (RBE4)

The immortalized rat brain endothelial cell line (RBE4) shows typical endothelial morphology and retains many brain endothelial cell characteristics [53,54,55,56,57]. RBE4 cells, kindly provided by Michael Aschner (Department of Pediatrics, Vanderbilt Kennedy Centre, Nashville, TN, USA), were plated on collagen-coated dishes or flasks (50 μg/mL in acetic acid 0.02 M). Cells were grown at 37 °C in a 5% CO_2_ atmosphere in the presence of alpha-MEM/F-10 Nutrient medium (1:1) supplemented with 10% heat-inactivated fetal bovine serum (FBS) 1% penicillin and streptomycin, 1% glutamine, and 300 ug/mL Geneticin. RBE4 were plated in collagen-coated 96 multiwells (6 × 10^3^ cells/well) for ROS and MTT assays, while cells were plated in 35 mm diameter Petri dishes (8 × 10^4^ cells/well) for protein evaluation. Twenty-four hours after plating, RBE4 cells were treated or not with coffee metabolites or coffee pulp phytoextract and maintained in culture a further 24 h before pro-oxidant exposure.

### 3.3. Coffee Phenolic Metabolites

The coffee phenolic metabolites tested in this study were chosen among the ones detected in the plasma of individuals drinking coffee daily [16]: dihydrocaffeic acid, dihydroferulic acid, dihydroferulic acid-4′-sulfate and ferulic acid-4′-sulfate (mainly methylated and sulfated compounds), and caffeic acid, caffeic acid-3′-glucuronide, caffeic acid-4′-glucuronide, and dihydrocaffeic acid-3′-glucuronide (no methylated, glucuronidated compounds). A schematic representation of coffee metabolites as published in [35]. In order to mimic a more physiological scenario, the metabolites were evaluated at a concentration of 100 nM, alone or blended all together in a mix. Since these substances had to be dissolved in DMSO before being diluted in a DMEM medium, the effects of DMSO concentrations on cell viability were also assayed.

### 3.4. Coffee Pulp Extraction Process and LC-MS Analysis

The coffee pulp phytoextract (Phyt) derives from pulp coffee waste processing of an agricultural cooperative in El Salvador, which cultivates the variety Pacas of *Coffea* *arabica* L. species. Phytoextract (used in this work was obtained by a 100% aqueous maceration as previously described [22]. The freeze-dried extract was stored at −20 °C prior to chemical and biological analysis. Qualitative on-line liquid chromatography high-resolution (HR) mass spectrometry (MS) analysis of extracts was performed using a Shimadzu Ultra High-Pressure Liquid Chromatograph (UHPLC) Nexera system (Kyoto, Japan) interfaced to a TripleTOF^®^ 6600 System with a DuoSpray™ Source (SCIEX; Foster City, CA, USA). For details, see Magoni et al. [22]. The phenolic compounds detected are listed in Table 1.

To evaluate the viability and antioxidant properties, lyophilized samples were solubilized in the medium at a concentration of 10 mg/mL and subsequently were diluted to working concentrations of 100–500 µg/mL.

### 3.5. Tert-Butyl Hydroperoxide (TBHP) Treatment

*Tert*-butyl hydroperoxide (TBHP) is commonly used to induce oxidative stress in order to mimic intracellular ROS increase during a pathological condition [58]. The pro-oxidant treatment condition was chosen after a dose-dependent (50–500 µM) assessment of viability and ROS production in RBE4 cells. The concentration of 200 µM TBHP resulted in maximum ROS levels without adversely affecting viability (data not shown). TBHP was diluted to a final concentration of 200 µM in medium and administered to cells pretreated or not with coffee metabolites or pulp Phyt.

### 3.6. Cell Viability Analysis

After coffee metabolites or phytoextract treatments, cell viability was evaluated by means of MTT (3-[4,5-dimethylthiazol-2-yl]-2,5-diphenyl-2*H*-tetrazolium bromide) assay. MTT is uptaken by cells and transformed into formazan by mitochondrial succinate dehydrogenase. Accumulation of formazan directly reflects the activity of mitochondria as an indirect measurement of cell viability. MTT stock solution (5 mg/mL) was added to each plate to a final concentration of 1.2 mM, and cells were incubated for 1.5 h at 37 °C. After removing the MTT solution, the reaction was stopped by adding EtOH. Resuspended cells were centrifuged for 10 min at 800× *g*, the absorbance was measured at a wavelength of 560 nm and a reference wavelength of 690 nm using Victor3 1420 Multilabel Counter (Perkin Elmer, Waltham, MA, USA), and the percentage viability was calculated.

### 3.7. Determination of Intracellular Reactive Oxygen Species (ROS)

Intracellular ROS production was estimated by using 2,7-dichlorofluorescein diacetate (DCF-DA) as a probe [59]. DCF-DA diffuses through the cell membrane, where it is enzymatically deacetylated by intracellular esterases to the more hydrophilic nonfluorescent reduced dye dichlorofluorescein. In the presence of reactive oxygen species, nonfluorescent DCFH rapidly oxidized to highly fluorescent product DCF. Based on the method setup, after performing the experiments (as described below), RBE4 cells were incubated with a 10 μM DCFH-DA probe in a serum-free medium for 1 h at 37 °C. The formation of DCF was measured at the excitation wavelength of 485 nm and an emission wavelength of 535 nm using a fluorescence spectrometer (Tecan Infinite^®^ M200 Pro, Männedorf, Switzerland). ROS production was normalized as a percentage of control.

### 3.8. Oxygen and Glucose Deprivation (OGD) Treatment

Cells were subjected to oxygen and glucose deprivation (OGD), and subsequent restoration of normoxic and normoglucidic conditions (ogR) as previously described [60]. Briefly, the culture medium was replaced with a glucose-free balanced salt solution (BSS) with or without metabolites or phytoextract, and then cells were incubated at 37 °C for 3 h in a hypoxia chamber (Billups–Rothenberg, San Diego, CA, USA) saturated for 10 min with 5% CO_2_: 95% N_2_ and sealed. Afterward, OGD, normoxic, and normoglycemic conditions were restored for 1 h or 24 h. Cells were replaced in normal culture conditions (37 °C in a 5% CO_2_ atmosphere), adding in each dish a restoration solution containing 5 mM glucose and 10% FBS in the culture medium. From here on, we will use the term “oxygen and glucose Restoration” (ogR) to indicate “normoxic and normoglycemic conditions restoration post-OGD”. RBE4 untreated cells were maintained in normal culture conditions and collected together with treated cells subjected to ogR.

### 3.9. Cell Fractionation

RBE4 cells treated or not with coffee metabolites or phytoextract for 24 h and then subjected to OGD/ogR were washed and harvested in 2 mL of PBS plus protease inhibitors and centrifuged at 4 °C for 5 min at 1000 rpm. The cellular pellet was resuspended in 250 µL of a commercial buffer for nuclei extraction (Nuclei EZ Lysis Buffer, Sigma-Aldrich, Milano, Italy), containing phosphatase and protease inhibitors, at 4 °C for 5 min. The homogenate was then centrifuged at 550× *g* for 10 min at 4 °C to separate the nuclear fraction from the cytoplasmic fraction. The nuclear fraction was resuspended in 250 µL of denaturation buffer and boiled for 5 min at 100 °C. The nuclear fraction and the cytoplasmic fraction of each sample were analyzed by SDS PAGE electrophoresis and Western blotting.

### 3.10. SDS-PAGE and Immunoblotting

Protein analysis was performed by SDS-PAGE electrophoresis on 10% polyacrylamide tris-glycine, protein transfer to a nitrocellulose membrane (Amersham, GE Healthcare Europe GmbH, Milano, Italy), revelation by Ponceau staining (Sigma Chemical Co., Milano, Italy), and immunoblotting with specific antibodies. Electrophoresis separation and Western blotting were carried out on equal amounts of homogenate in order to investigate protein level expression in cellular membranes. Nitrocellulose membranes were blocked in TBS-Tween 0.1% or 0.2% buffer containing 5% non-fat milk or 5% BSA and probed with specific antibodies diluted in the same solution. Immunoblotting was performed using anti-Akt (1:1000), anti-P-Akt (1:1000), anti-Erk 1/2 (1:1000), anti-P-Erk 1/2 (1:1000), anti-HO-1 (1:200), anti-Nrf2 (1:250), anti-β-actin (1:1500), anti-LDH (1:1000), and anti-LaminB (1:1000). Immunoreactive proteins were revealed by ECL and semiquantitatively estimated by LAS4000 Image Station (GE Healthcare Italia, Milano, MI, Italy). Normalization in the same sample was carried out with respect to β-actin. In the analysis for Nrf2 localization, laminB and LDH were used to normalize nuclear Nrf2 and cytosolic Nrf2, respectively.

### 3.11. Statistic Analysis

Biochemical determinations were obtained from at least three independent experiments. All data are presented as mean ± SE. Statistical differences were tested by one-way ANOVA and Student’s *t*-test. A difference was considered significant at the 95% level (*p* < 0.05).

## 4. Conclusions

In summary, the coffee pulp phytoextract attenuates ROS accumulation, acting as a free radical scavenger and inducing Nrf2-driven endogenous antioxidant defenses. Probably, its antioxidant power depends on the presence of procyanidins and quinic acid derivatives working in synergy. This is in line with a recent study demonstrating that coffee bean extracts reduced the oxidative damage under I/R through the activation of Nrf2 signaling [61], adding the valuable concept of waste biomass reuse as a source of antioxidant compounds. As the results showed, the different phenolics contained in the Phyt determined specific antioxidant properties able to counteract the oxidative stress generated during the reperfusion, suggesting that the intake of coffee-derived Phyt as a dietary supplement might represent a nutritional strategy to counteract age-related damage. It has been hypothesized that the consumption of a relatively low amount, ~2 mg, of coffee pulp extract [22], could be sufficient to get the effective antioxidant dosage. Nevertheless, further studies are needed to quantify the real extract amount to reach, after digestion, a sufficient polyphenol concentration to activate the antioxidant defence shown in the current research. In the future, it will be possible to develop a functional food formulation containing coffee pulp phytoextract. Then, a repeated dose, randomized, human intervention in a group of volunteers could be performed to evaluate the impact of functional food daily intake on health. Moreover, the use of waste biomass could have a positive impact on local ecosystems, reducing the pollution of the soil and water. It could also contribute to improving the producing countries’ economy, which is often based only on the sale of coffee seeds.

## Figures and Tables

**Table 1:** Components of phytoextract identified by mass spectrometry (LC-MS).

Peak Number	Compound
1	Caffeoylquinic acid
2	*p*-coumaroylquinic acid
3	Caffeoylquinic acid
4	Caffeoylquinic acid
5	Feruloylquinic acid
6	Caffeoylquinic acid
7	*p*-coumaroylquinic acid
8	Feruloylquinic acid
9	Feruloyl quinic acid
10	*p*-coumaroylquinic acid
11	Feruloylquinic acid
12	Di-caffeoylquinic acid
13	Di-caffeoylquinic acid
14	Di-caffeoylquinic acid
15	Di-caffeoylquinic acid
16	3-*O-p*-coumaroyl-4-*O*-caffeoylquinic acid
17	3-*O*-feruloyl-4-*O*-caffeoylquinic acid
18	3-*O*-caffeoyl-4-*O*-*p*-coumaroylquinic acid
19	3-*O*-caffeoyl-4-*O*-feruloylquinic acid
20	4-*O*-caffeoyl-5-*O-p*-coumaroyl quinic acid
21	3-*O*-feruloyl-5-*O*-caffeoylquinic acid
22	3-*O*-caffeoyl-5-*O*-feruloylquinic acid
23	4-*O*-feruloyl-5-*O*-caffeoylquinic acid
24	4-*O*-caffeoyl-5-*O*-feruloylquinic acid

## Data Availability

The datasets used and analyzed during the current study are available from the corresponding author on reasonable request.

## Sample Availability

Samples of the compounds are available from the authors.

## References

[1] Sies H., Berndt C., Jones D.P. (2017). Oxidative Stress. Annu. Rev. Biochem..

[2] Sies H., Sies H. (1985). Introductory Remarks. Oxidative Stress.

[3] Cenini G., Lloret A., Cascella R. (2019). Oxidative Stress in Neurodegenerative Diseases: From a Mitochondrial Point of View. Oxidative Med. Cell. Longev..

[4] Lin D., Wang L., Yan S., Zhang Q., Zhang J.H., Shao A. (2019). The Role of Oxidative Stress in Common Risk Factors and Mechanisms of Cardio-Cerebrovascular Ischemia and Depression. Oxidative Med. Cell. Longev..

[5] Verhoeven J.I., Allach Y., Vaartjes I.C.H., Klijn C.J.M., de Leeuw F.E. (2021). Ambient air pollution and the risk of ischaemic and haemorrhagic stroke. Lancet Planet. Health.

[6] Guo M., Lu H., Qin J., Qu S., Wang W., Guo Y., Liao W., Song M., Chen J., Wang Y. (2019). Biochanin A Provides Neuroprotection Against Cerebral Ischemia/Reperfusion Injury by Nrf2-Mediated Inhibition of Oxidative Stress and Inflammation Signaling Pathway in Rats. Med. Sci. Monit..

[7] Panuganti K.K., Tadi P., Lui F. (2021). Transient Ischemic Attack.

[8] Suardi C., Cazzaniga E., Graci S., Dongo D., Palestini P. (2021). Link between Viral Infections, Immune System, Inflammation and Diet. Int. J. Environ. Res. Public Health.

[9] Yao J., Peng S., Xu J., Fang J. (2019). Reversing ROS-mediated neurotoxicity by chlorogenic acid involves its direct antioxidant activity and activation of Nrf2-ARE signaling pathway. BioFactors.

[10] Ames B.N. (2018). Prolonging healthy aging: Longevity vitamins and proteins. Proc. Natl. Acad. Sci. USA.

[11] Pham-Huy L.A., He H., Pham-Huy C. (2008). Free radicals, antioxidants in disease and health. Int. J. Biomed. Sci. IJBS.

[12] Visioli F., De La Lastra C.A., Andres-Lacueva C., Aviram M., Calhau C., Cassano A., D’Archivio M., Faria A., Favé G., Fogliano V. (2011). Polyphenols and human health: A prospectus. Crit. Rev. Food Sci. Nutr..

[13] International Coffee Organization What’s New. ico.org.

[14] Zamora-Ros R., Knaze V., Rothwell J.A., Hemon B., Moskal A., Overvad K., Tjonneland A., Kyro C., Fagherazzi G., Boutron-Ruault M.C. (2016). Dietary polyphenol intake in Europe: The European Prospective Investigation into Cancer and Nutrition (EPIC) study. Eur. J. Nutr..

[15] Romualdo G.R., Rocha A.B., Vinken M., Cogliati B., Moreno F.S., Chaves M.A.G., Barbisan L.F. (2019). Drinking for protection? Epidemiological and experimental evidence on the beneficial effects of coffee or major coffee compounds against gastrointestinal and liver carcinogenesis. Food Res. Int..

[16] Mena P., Bresciani L., Tassotti M., Rosi A., Martini D., Antonini M., Cas A.D., Bonadonna R., Brighenti F., Del Rio D. (2021). Effect of different patterns of consumption of coffee and a cocoa-based product containing coffee on the nutrikinetics and urinary excretion of phenolic compounds. Am. J. Clin. Nutr..

[17] Stalmach A., Mullen W., Barron D., Uchida K., Yokota T., Cavin C., Steiling H., Williamson G., Crozier A. (2009). Metabolite profiling of hydroxycinnamate derivatives in plasma and urine after the ingestion of coffee by humans: Identification of biomarkers of coffee consumption. Drug Metab. Dispos..

[18] Mussatto S.I., Machado E.M.S., Martins S., Teixeira J.A. (2011). Production, composition and application of coffee and its industrial residues. Food Bioprocess Technol..

[19] Cristobal J., Matos C.T., Aurambout J.P., Manfredi S., Kavalov B. (2016). Environmental sustainability assessment of bioeconomy value chains. Biomass Bioenergy.

[20] Da Silva M.R., Bragagnolo F.S., Carneiro R.L., Pereira I.D.O.C., Ribeiro J.A.A., Rodrigues C.M., Jelley R.J., Fedrizzi B., Funari C.S. (2021). Metabolite characterization of fifteen by-products of the coffee production chain: From farm to factory. Food Chem..

[21] Lachenmeier D.W., Schwarz S., Rieke-Zapp J., Cantergiani E., Rawel H., Martín-Cabrejas M.A., Martuscelli M., Gottstein V., Angeloni S. (2021). Coffee By-Products as Sustainable Novel Foods: Report of the 2nd International Electronic Conference on Foods—“Future Foods and Food Technologies for a Sustainable World”. Foods.

[22] Magoni C., Bruni I., Guzzetti L., Dell’Agli M., Sangiovanni E., Piazza S., Regonesi M.E., Maldini M., Spezzano R., Caruso D. (2018). Valorizing coffee pulp by-products as anti-inflammatory ingredient of food supplements acting on IL-8 release. Food Res. Int..

[23] Cao X., Yang L., Xue Q., Yao F., Sun J., Yang F., Liu Y. (2020). Antioxidant evaluation-guided chemical profiling and structure-activity analysis of leaf extracts from five trees in Broussonetia and Morus (Moraceae). Sci. Rep..

[24] Valencia-Hernandez L.J., Wong-Paz J.E., Ascacio-Valdés J.A., Chávez-González M.L., Contreras-Esquivel J.C., Aguilar C.N. (2021). Procyanidins: From Agro-Industrial Waste to Food asBioactive Molecules. Foods.

[25] Morris M.C., Evans D.A., Tangney C.C., Bienias J.L., Wilson R.S. (2006). Associations of vegetable and fruit consumption with age-related cognitive change. Neurology.

[26] Nooyens A.C., Bueno-de-Mesquita H.B., van Boxtel M.P., van Gelder B.M., Verhagen H., Verschuren W.M. (2011). Fruit and vegetable intake and cognitive decline in middle-aged men and women: The Doetinchem Cohort Study. Br. J. Nutr..

[27] Ricci A., Parpinello G.P., Versari A. (2018). The Nutraceutical Impact of Polyphenolic Composition in Commonly Consumed Green Tea, Green Coffee and Red Wine Beverages: A Review. Recent Adv. Food Sci. Nutr. Res..

[28] Adibhatla R.M., Dempsy R., Hatcher J.F. (2008). Integration of cytokine biology and lipid metabolism in stroke. Front. Biosci..

[29] Liang G., Shi B., Luo W., Yang J. (2015). The protective effect of caffeic acid on global cerebral ischemia-reperfusion injury in rats. Behav. Brain Funct..

[30] Zhou Y., Fang S.H., Ye Y.L., Chu L.S., Zhang W.P., Wang M.L., Wei E.Q. (2006). Caffeic acid ameliorates early and delayed brain injuries after focal cerebral ischemia in rats. Acta Pharmacol. Sin..

[31] Olthof M.R., Hollman P.C., Katan M.B. (2001). Chlorogenic acid and caffeic acid are absorbed in humans. J. Nutr..

[32] Del Rio D., Stalmach A., Calani L., Crozier A. (2010). Bioavailability of coffee chlorogenic acids and green tea flavan-3-ols. Nutrients.

[33] Martini D., Del Bo C., Tassotti M., Riso P., Del Rio D., Brighenti F., Porrini M. (2016). Coffee consumption and oxidative stress: A review of human intervention studies. Molecules.

[34] Li C., Jackson R.M. (2002). Reactive species mechanisms of cellular hypoxia-reoxygenation injury. Am. J. Physiol. Cell Physiol..

[35] Botto L., Bulbarelli A., Lonati E., Cazzaniga E., Tassotti M., Mena P., Del Rio D., Palestini P. (2021). Study of the Antioxidant Effects of Coffee Phenolic Metabolites on C6 Glioma Cells Exposed to Diesel Exhaust Particles. Antioxidants.

[36] Upadhyay R., Mohan Rao L.J. (2013). An outlook on chlorogenic acids-occurrence, chemistry, technology, and biological activities. Crit. Rev. Food Sci. Nutr..

[37] Duangjai A., Suphrom N., Wungrath J., Ontawong A., Nuengchamnong N., Yosboonruang A. (2016). Comparison of antioxidant, antimicrobial activities and chemical profiles of three coffee (*Coffea arabica* L.) pulp aqueous extracts. Integr. Med. Res..

[38] Kamdem J., Waczuk E., Kade I., Wagner C., Boligon A., Athayde M., Souza D. (2012). Catuaba (*Trichilia catigua*) prevents against oxidative damage induced by in vitro ischemia-reperfusion in rat hippocampal slices. Neurochem. Res..

[39] Ya B.L., Liu Q., Li H.F., Cheng H.J., Yu T., Chen L., Wang Y., Yuan L.L., Li W.J., Liu W.Y. (2018). Uric Acid Protects against Focal Cerebral Ischemia/Reperfusion-Induced Oxidative Stress via Activating Nrf2 and Regulating Neurotrophic Factor Expression. Oxidative Med. Cell. Longev..

[40] Wang L., Chen Y., Sternberg P., Cai J. (2008). Essential roles of the PI3 kinase/Akt pathway in regulating Nrf2-dependent antioxidant functions in the RPE. Investig. Opthalmology Vis. Sci..

[41] Zipper L.M., Mulcahy R.T. (2003). Erk activation is required for Nrf2 nuclear localization during pyrrolidine dithiocarbamate induction of glutamate cysteine ligase modulatory gene expression in HepG2 cells. Toxicol Sci..

[42] Owuor E.D., Kong A.N. (2002). Antioxidants and oxidants regulated signal transduction pathways. Biochem. Pharm..

[43] Soares R., Losada D., Jordani M., Évora P. (2019). Ischemia/Reperfusion Injury Revisited: An Overview of the Latest Pharmacological Strategies. Int. J. Mol. Sci..

[44] Lonati E., Corsetto P.A., Montorfano G., Zava S., Carrozzini T., Brambilla A., Botto L., Palestini P., Rizzo A.M., Bulbarelli A. (2019). Lipid reshaping and lipophagy are induced in a modeled Ischemia-Reperfusion injury of Blood Brain Barrier. Int. J. Mol. Sci..

[45] Buscà R., Pouysségur J., Lenormand P. (2016). ERK1 and ERK2 Map Kinases: Specific Roles or Functional Redundancy?. Front. Cell Dev. Biol..

[46] Kim J.K., Jang H.D. (2014). Nrf2-mediated HO-1 induction coupled with the ERK signaling pathway contributes to indirect antioxidant capacity of caffeic acid phenethyl ester in HepG2 cells. Int. J. Mol. Sci..

[47] Ryter S.W., Choi A.M. (2005). Heme oxygenase-1: Redox regulation of a stress protein in lung and cell culture models. Antioxid. Redox Signal..

[48] Han D., Chen W., Gu X., Shan R., Zou J., Liu G., Shahid M., Gao J., Han B. (2017). Cytoprotective effect of chlorogenic acid against hydrogen peroxide-induced oxidative stress in MC3T3-E1 cells through PI3K/Akt-mediated Nrf2/HO-1 signaling pathway. Oncotarget.

[49] Yu Y., Li Z., Cao G., Huang S., Yang H. (2019). Bamboo Leaf Flavonoids Extracts Alleviate Oxidative Stress in HepG2 Cells via Naturally Modulating Reactive Oxygen Species Production and Nrf2-Mediated Antioxidant Defense Responses. J. Food Sci..

[50] Chen X., Yang J.H., Cho S.S., Kim J.H., Xu J., Seo K., Ki S.H. (2020). 5-Caffeoylquinic acid ameliorates oxidative stress-mediated cell death via Nrf2 activation in hepatocytes. Pharm. Biol..

[51] Wang Q.Q., Gao H., Yuan R., Han S., Li X.X., Tang M., Dong B., Li J.X., Zhao L.C., Feng J. (2020). Procyanidin A2, a polyphenolic compound, exerts anti-inflammatory and anti-oxidative activity in lipopolysaccharide-stimulated RAW264.7 cells. PLoS ONE.

[52] Han S., Gao H., Chen S., Wang Q., Li X., Du L.J., Li J., Luo Y.Y., Li J.X., Zhao L.C. (2019). Procyanidin A1 Alleviates Inflammatory Response induced by LPS through NF-κB, MAPK, and Nrf2/HO-1 Pathways in RAW264.7 cells. Sci. Rep..

[53] Aschner M., Fitsanakis V.A., dos Santos A.P., Olivi L., Bressler J.P. (2006). Blood-brain barrier and cell-cell interactions: Methods for establishing in vitro models of the blood-brain barrier and transport measurements. Methods Mol. Biol..

[54] Balbuena P., Li W., Ehrich M. (2011). Assessments of tight junction proteins occludin, claudin 5 and scaffold proteins ZO1 and ZO2 in endothelial cells of the rat blood-brain barrier: Cellular responses to neurotoxicants malathion and lead acetate. Neurotoxicology.

[55] Faria A., Pestana D., Teixeira D., Couraud P.O., Romero I., Weksler B., de Freitas V., Mateus N., Calhau C. (2011). Insights into the putative catechin and epicatechin transport across blood-brain barrier. Food Funct..

[56] Roux F., Couraud P.O. (2005). Rat brain endothelial cell lines for the study of blood-brain barrier permeability and transport functions. Cell. Mol. Neurobiol..

[57] Wilhelm I., Fazakas C., Krizbai I.A. (2011). In vitro models of the blood-brain barrier. Acta Neurobiol. Exp..

[58] Robb S.J., Connor J.R. (1998). An in vitro model for analysis of oxidative death in primary mouse astrocytes. Brain Res..

[59] Hansen M.B., Nielsen S.E., Berg K. (1989). Re-examination and further development of a precise and rapid dye method for measuring cell growth/cell kill. J. Immunol. Methods.

[60] Bulbarelli A., Lonati E., Brambilla A., Orlando A., Cazzaniga E., Piazza F., Ferrarese C., Masserini M., Sancini G. (2012). Aβ42 production in brain capillary endothelial cells after oxygen and glucose deprivation. Mol. Cell. Neurosci..

[61] Rizk S., Taha H., Abdel Moneim A.E., Amin H.K. (2021). Neuroprotective effect of green and roasted coffee bean extracts on cerebral ischemia-induced injury in rats. Metab. Brain Dis..

